# Renin and angiotensinogen expression and functions in growth and apoptosis of human glioblastoma

**DOI:** 10.1038/sj.bjc.6601646

**Published:** 2004-03-02

**Authors:** L Juillerat-Jeanneret, J Celerier, C Chapuis Bernasconi, G Nguyen, W Wostl, H P Maerki, R-C Janzer, P Corvol, J-M Gasc

**Affiliations:** 1 University Institute of Pathology, CHUV, Bugnon 25, Lausanne CH1011, Switzerland; 2 INSERM U36, Collège de France, 10 place M. Berthelot, Paris F75005, France; 3 INSERM U489, Hôpital Tenon, 4 rue de la Chine, Paris F75020, France; 4 Hoffmann-La Roche Ltd, Pharmaceuticals Division, Basel CH 4070, Switzerland

**Keywords:** renin, angiotensinogen, glioblastoma, renin inhibitors, apoptosis, human

## Abstract

The expression and function in growth and apoptosis of the renin–angiotensin system (RAS) was evaluated in human glioblastoma. Renin and angiotensinogen (AGT) mRNAs and proteins were found by *in situ* hybridisation and immunohistochemistry in glioblastoma cells. Angiotensinogen was present in glioblastoma cystic fluids. Thus, human glioblastoma cells produce renin and AGT and secrete AGT. Human glioblastoma and glioblastoma cells expressed renin, AGT, renin receptor, AT_2_ and/or AT_1_ mRNAs and proteins determined by RT–PCR and/or Western blotting, respectively. The function of the RAS in glioblastoma was studied using human glioblastoma cells in culture. Angiotensinogen, des(Ang I)AGT, tetradecapaptide renin substrate (AGT1–14), Ang I, Ang II or Ang III, added to glioblastoma cells in culture, did not modulate their proliferation, survival or death. Angiotensin-converting enzyme inhibitors did not diminish glioblastoma cell proliferation. However, the addition of selective synthetic renin inhibitors to glioblastoma cells decreased DNA synthesis and viable tumour cell number, and induced apoptosis. This effect was not counterbalanced by concomitant addition of Ang II. In conclusion, the complete RAS is expressed by human glioblastomas and glioblastoma cells in culture. Inhibition of renin in glioblastoma cells may be a potential approach to control glioblastoma cell proliferation and survival, and glioblastoma progression in combination therapy.

In addition to their functions in controlling vascular tone and natriohydric balance, the components of the renin–angiotensin system (RAS) may be involved in cell growth and survival. Angiotensin peptides may promote cell growth, whereas angiotensinogen (AGT) was shown to inhibit vascular cell growth and angiogenesis (Célérier *et al*, 2002). These functions may be of particular importance in human disorders related to growth dysfunctions, either increased proliferation such as cardiovascular remodelling and cancer, or diminished proliferation such as defects in tissue repair.

The RAS is composed of a precursor molecule, AGT, a peptide related to the family of serine protease inhibitors (serpins), as the unique substrate for the protease renin (EC 3.4.23.15). The hydrolysis of AGT by renin is rate-limiting for the whole system and results in the production of des(Ang I)-AGT and of the vasoinactive peptide Ang I, which is converted to the vasoactive peptides Ang II and Ang III by angiotensin-converting enzyme (ACE) (EC 3.4.15.1) and aminopeptidase A (EC 3.4.11.7), respectively. These peptides act on two membrane-bound receptors (AT_1_ and AT_2_) belonging to the seven-transmembrane G-protein-coupled receptor (GPCR) family. More recently, it has been suggested that, in addition to the release of Ang I from AGT, renin might act directly on cell function as a ligand for a cell-membrane receptor, resulting in the phosphorylation of ERK (extracellular regulated kinase) (Takahashi *et al*, 1985; Nguyen *et al*, 1996, 2002). Inhibitors of the renin and ACE enzymes as well as antagonists to the AT_1_ receptors have been developed in the context of cardiovascular disorders.

The RAS is expressed independently of the circulating RAS in normal nonvascular tissues (Inwang *et al*, 1997; Tahmasebi *et al*, 1998; Hirasawa *et al*, 2002), including the CNS (Milsted *et al*, 1990; Humpel *et al*, 1994), where, in addition to regulating cardiovascular functions (Morimoto *et al*, 2002), its role may include the control of cell death and/or growth (Kakinuma *et al*, 1997). Renin-secreting tumours of several nonrenal origins have been described and the role of the RAS has been evaluated in a few situations in human cancer. In breast cancer, Ang II increased integrin expression (Berry *et al*, 2001) and Ang II exerted growth-promoting effects via the AT_1_ receptor (De Paepe *et al*, 2001). In a C6 glioblastoma rat model, losartan (AT_1_ antagonist) reduced tumour growth, vascular density, cell proliferation and mitotic index (Rivera *et al*, 2001). We have previously shown high expression of ACE protein in human glioblastoma vessels; however, inhibition of ACE did not modify the growth of an experimental glioblastoma implanted in rat brains (Juillerat-Jeanneret *et al*, 2000). The production of renin and AGT or a potential function of renin, angiotensins and/or AGT in these tumours was not evaluated in these previous experiments. Therefore, in the present study, we report the expression and functions of renin, AGT and angiotensin peptides in human glioblastoma tumours and human glioblastoma cells in culture.

## MATERIAL AND METHODS

### Human surgical specimens

Human astrocytoma and glioblastoma samples (grade II, *n*=1, grade III, *n*=6; grade IV, *n*=5) were retrospectively selected from surgical diagnostic biopsies or subtotal resection specimens, either fixed in 4% buffered paraformaldehyde and embedded in paraffin, or frozen in liquid nitrogen and stored at −80°C. Paraffin-embedded samples were used for immunohistochemistry and *in situ* hybridisation and frozen samples for Western blotting and RT–PCR experiments. The fluid content of glioblastoma pseudocysts (due to the absence of epithelial lining, the term cyst cannot be used formally) was obtained at the time of surgery by aspiration of the fluid, and stored at −80°C. Plasma was obtained from patients with various diseases and cerebrospinal fluids were retrospectively selected from patients with brain tumours undergoing punctions for diagnostic purposes.

### Immunohistochemistry

Paraffin-embedded sections (5 *μ*m thick) of human glioblastoma were deparaffinised in xylene and isopropanol, and endogenous peroxidase was inactivated in 3% hydrogen peroxide in methanol. Sections were incubated with the antirenin 2D12/F37 and 4G1/F55 monoclonal antibodies, as previously described (Galen *et al*, 1984; Juillerat-Jeanneret *et al*, 2000), anti-AGT (N-1345 and C-1350 (Célérier *et al*, 2000)), anti-des(Ang I)-AGT (D854 (Célérier *et al*, 2000)), and subsequently exposed to peroxidase-conjugated secondary immunoglobulins. Peroxidase activity was visualised using 0.035% diaminobenzidine (Fluka) as a chromogen, and slides were counterstained with haematoxylin.

### *In situ* hybridisation

*In situ* hybridisation for renin, ACE and AGT was performed essentially as previously described (Sibony *et al*, 1995). Briefly, paraffin sections (7 *μ*m) were deparaffinised, rehydrated, heated in a microwave oven, and digested with proteinase K (Roche Diagnostics) before hybridisation with the ^35^S-labelled riboprobes (3–4 × 10^5^ cpm section^−1^). Hybridisation was performed overnight at 50°C and was followed by several washes and an RNAse treatment to remove single-strand nonhybridised cRNA strands. Sense probes were used as controls. Sections were exposed in the dark, processed for autoradiography and counterstained with toluidine blue. Observation was performed under dark field or bright field illumination.

### AGT concentration measurements

The concentration of AGT in glioblastoma ascitic fluids was determined by measuring the production of Ang I concentrations at 37°C in the absence or presence of added renin, as previously described (Célérier *et al*, 2002). AGT equivalents were calculated from the Ang I generated from AGT present in the ascitic fluid.

### RT–PCR and Western blotting determination of the components of the RAS

Total RNA was isolated from cells in culture or from surgical samples using the Trizol reagent (Gibco-BRL, Basel, Switzerland). RT–PCR was performed according to standard procedures and 35 cycles, using primer sequences given in Table 1

**Table 1:** Specific primers for the amplification of renin, renin receptor, angiotensinogen, AT_1_ and AT_2_

**Gene**	**Primer sequences for RT–PCR**	**Size of amplified fragments**
Renin	Sense 5′-GTG TCT GTG GGG TCA TCC ACC TTG-3′	
	Antisense 5′-GGA TTC CTG AAA TAC ATA GTC CGT-3′	243 bp
	Renin-receptor sense 5′-TGT TTT GGG GAA CGA GTT TAG TA-3	
	Antisense 5′-GAG CGT CAA CAA GGA TCT TAG AA-3′	691 bp

AGT	Sense 5′-TCC ACC TCG TCA TCC ACA-3′	
	Antisense 5′-GGC TCC CAG ATA GAG AGA-3′	329 bp

AT1	Sense 5′-CTT TTC CTG GAT TCC CCA C-3′	
	Antisense 5′-CTT CTT GGT GGA TGA GCT TAC-3′	304 bp

AT2	Sense 5′-GTG ACC AAG TCC TGA AGA TG-3′	
	Antisense 5′-CAC AAA GGT CTC CAT TTC TC-3′	335 bp

AGT: angiotensinogen; AT: angiotensin receptor.

. Positive controls were mRNAs extracted from normal human kidney, liver, pulmonary artery or breast cancer. As controls for RNA quality, amplification reactions were performed using pairs of primers specific for glyceraldehyde-3-phosphate dehydrogenase (GAPDH; Egidy *et al*, 2000). Amplified transcripts were analysed on 2% agarose gels.

Proteins were extracted from confluent layers of glioblastoma cell cultures in 40 mM HEPES pH 7.5 containing 100 mM NaCl, 0.1 mM EDTA, 100 mM PMSF, 10% glycerol, 0.5% NP40, and the extracts were submitted to electrophoresis. After transfer, the membranes were probed using the F37 antirenin monoclonal antibody (Galen *et al*, 1984), a kind gift of Sanofi-Pasteur, Montpellier, polyclonal antirenin-receptor antibody (Nguyen *et al*, 2002), polyclonal anti-AT_1_ and anti-AT_2_ (Santa-Cruz Biotechnologies, Santa-Cruz, CA, USA) antibodies, and revealed using the ECL detection kit (Amersham, UK).

### Treatments of glioblastoma cell cultures

Human recombinant AGT or des(Ang I)AGT were obtained from AGT-transfected CHO-cell supernatants, as previously described (Célérier *et al*, 2002). CHO cells were grown in serum-free medium (Célérier *et al*, 2000) and secreted 15 mg AGT per litre of medium in 24 h. Mock-transfected cell supernatants were used as controls. Experiments were performed in the presence or absence of FasL-containing Neuro2A cell supernatants, as previously described (Egidy *et al*, 2000; Peduto Eberl *et al*, 2000). Human renin tetradecapeptide substrate (AGT1-14), Ang I, Ang II or Ang III were purchased from Bachem (Bubendorf, Switzerland) and dissolved in H_2_O. Pepstatin was purchased from Fluka (Buchs, Switzerland) and dissolved in methanol. Remikiren (Fischli *et al*, 1991) and (*R*)-3-[(3*S*,4*R*,5*R*)-4-[4-[3-(2-methoxy-benzyloxy)-propoxy]-phenyl]-5-(4-methoxy-naphthalen-2-ylmethoxy)-piperidin-3-ylmethoxy]-propane-1,2-diol (RO0663525} (Breu *et al*, 2000) were provided by Hoffmann-LaRoche (Basel, Switzerland). Stock solution of Remikiren was prepared in H_2_O and of RO0663525 in methanol, and then diluted in cell culture medium. The ACE inhibitors captopril (Sigma, Buchs, Switzerland) and lisinopril (a gift from Merck, Sharp and Dohme; Juillerat-Jeanneret, 1993) were dissolved at 10 mg ml^−1^ and diluted in culture medium. The AT_2_ antagonist PD123319 was purchased from Sigma. LN18 and LNZ308 human glioblastoma cells (a kind gift of AC Diserens, CHUV, Lausanne, Switzerland; Diserens *et al*, 1981) were grown in DMEM medium containing 4.5 g l^−1^ glucose and 5% FCS. Cells were split in 48-well plates and cultured for 1–3 days until confluence was reached. Peptides or inhibitors were added to the cells in culture for the time and concentration indicated. Then either 3-(4,5-dimethyl-2-thiazolyl)-2,5-diphenyl-2H-tetrazoliumbromide (MTT) reduction (cf below) to quantify the number of metabolically active viable cells, thymidine incorporation (cf below) to quantify DNA synthesis or measurement of nucleosome fragments to quantify apoptosis, were performed (cf below). Experiments were performed in triplicates at least three times and means±s.d. were calculated.

### Evaluation of cell viability, growth and apoptosis

The following techniques were performed as previously described: cell viability was determined using MTT (Sigma, Buchs, Switzerland) reduction and absorbance at 540 nm; DNA synthesis using [^3^H]-thymidine (0.2 *μ*Ci well^−1^; Amersham Pharmacia, Dübendorf, Switzerland) incorporation (Egidy *et al*, 2000) and apoptosis was quantified using the Cell Death Detection ELISA^PLUS^ (Roche, Rotkreuz, Switzerland), as previously described (Peduto Eberl *et al*, 2000).

### Protein concentration

Protein content was evaluated with the BCA protein assay kit (Pierce, Switzerland) according to the manufacturer's instructions, using bovine serum albumin as standard.

## RESULTS

**Figure 1:**
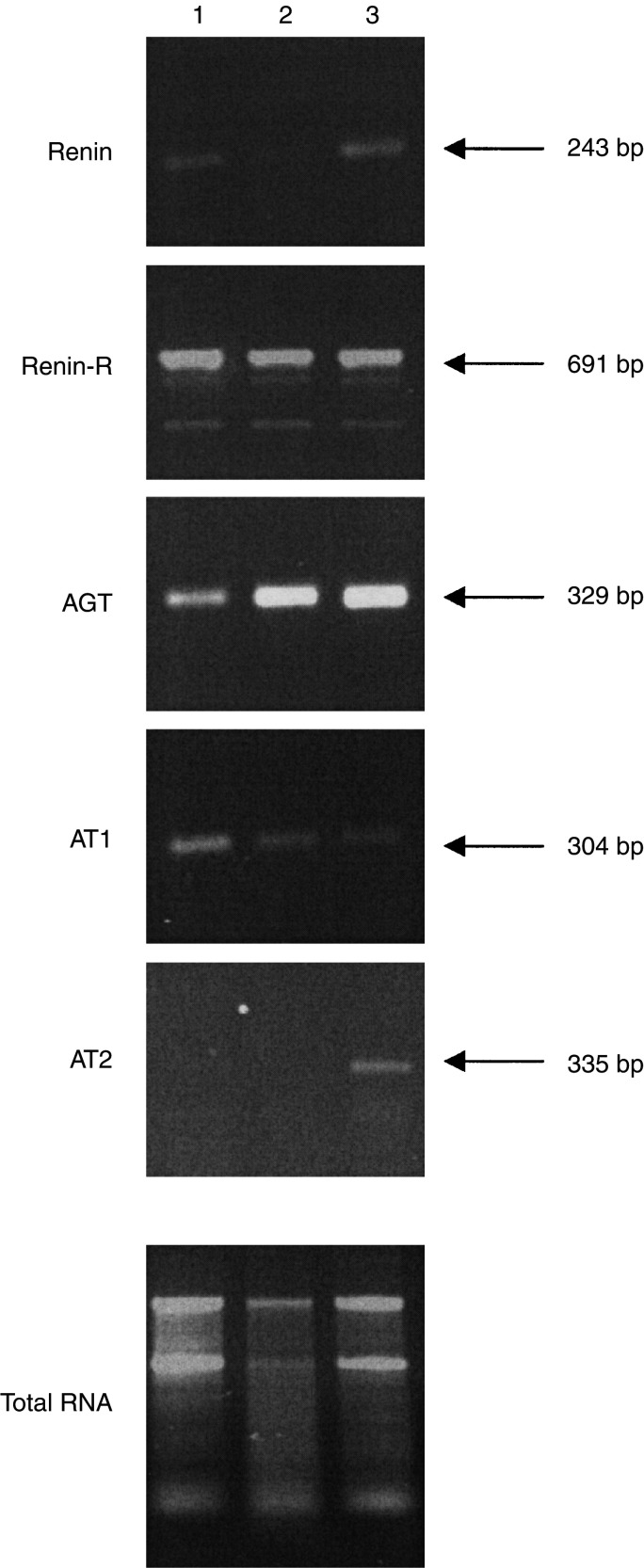
Determination of renin, renin receptor, AGT, AT_1_ and AT_2_ mRNAs by RT–PCR performed in three human glioblastoma surgical samples. RNA extracted from three human glioblastomas was used for RT–PCR analysis for renin, renin receptor (renin-R), AGT and angiotensin receptors (AT_1_ and AT_2_), using the specific primers described in Table 1.

**Figure 2:**
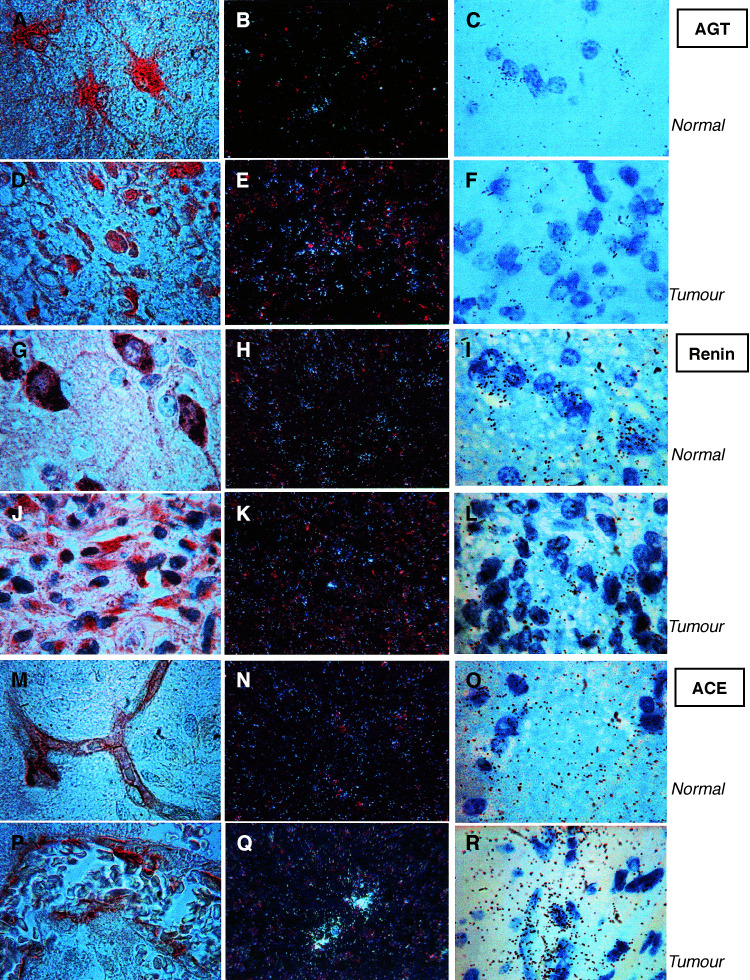
ISH and immunohistochemistry for renin, ACE and AGT in human glioblastoma and nontumoral associated tissue. Angiotensinogen (AGT) (**A–F**), renin (**G–L**) and angiotensin-converting enzyme (ACE) (**M–R**) expression in human glioblastoma (grade IV) (**D–F, J–L, P–R**) and nontumoral associated tissue (**A–C, G–I, M–O**). *In situ* hybridisation (ISH) (**B, C, E, F, H, I, K, L, N, O, Q, R**) was performed with the antisense probes for AGT, renin or ACE, and immunohistochemistry (**A, D, G, J, M, P**) with antibodies raised against human AGT, renin or ACE. Dark-field (**B, E, H, K, N, Q**) or bright-field illumination of the same tumour areas in consecutive slides. Sense probes or nonrelevant antibodies did not display any signal (not shown).

RT–PCR performed on three different glioblastoma surgical specimens demonstrated a heterogenous expression of the RAS components in human glioblastoma. All the three tumours expressed the mRNAs for renin receptor, AGT and AT_1_ (two at low level for this last molecule), two out of three expressed renin mRNA and one out of three AT_2_ mRNA (Figure 1

). The cellular localisation of AGT, renin and ACE synthesis in human glioblastoma was determined by *in situ* hybridisation (ISH) and immunohistochemistry (IHC) (Figure 2

). AGT mRNA and protein were expressed by nontumoral astrocytes and glioblastoma cells (Figure 2A–F). Renin mRNA and protein were highly expressed by nontumoral neurons (Figure 2G–I), macrophages (not shown; Juillerat-Jeanneret *et al*, 2000) and at a lower level by reactive astrocytes (not shown), and were nonhomogeneously expressed by tumour cells (Figure 2J–L). High ACE mRNA expression was found in tumour vessels (Figure 2Q, R), confirming our previous observation of a high expression of ACE protein in glioblastoma vasculature by immunohistochemistry (Juillerat-Jeanneret *et al*, 2000), but only at a very low level in the vessels of nontumoral tissue (Figure 2N, O). ACE protein was found in tumour-associated and non-tumoral vessels (Figure 2M, P).

In order to ascertain whether AGT was secreted by human tumours, its level was determined in human glioblastoma-associated pseudocyst fluid withdrawn at the time of surgery (Table 2

**Table 2:** Quantification of angiotensinogen in human glioblastoma pseudocyst

**Sample**	**Protein (mg ml^−1^)**	**AGT equivalent (*μ*g Ang I ml^−1^)**	**Specific activity (*μ*g AGT mg^−1^ prot)**
A-411	59.5	4.2	0.07
Gbl-747	53.3	4.2	0.08
Gbl-749	50.8	6.1	0.12
Gbl-859	52.8	4.7	0.09
Gbl-920	39.5	4.7	0.11
Gbl-988	59.7	5.2	0.09
Cystic fluids (*n*=6)	52.6±7.4	4.9±0.7	0.09±0.02

A: astrocytome grade II; Gbl: glioblastoma. Pseudocystic fluids (*n*=6) were incubated in the presence of added renin and Ang I produced was measured at the end of the incubation period, providing equivalent AGT. Mean±s.d. values of the combined samples were calculated.

). For the purpose of comparison, AGT in human cerebrospinal fluids (CSF) from various brain tumour patients (*n*=13, 0.17±0.10*μ*g AGT equivalent ml^−1^, 0.23±0.12 *μ*g AGT mg^−1^ protein) and in human plasma (*n*=12, 226.0±62.3*μ*g AGT equivalent ml^−1^, 3.23±1.22 *μ*g AGT mg^−1^ protein) was also measured. The approach used allowed to quantify Ang I release from AGT in the presence of added renin and protease inhibitors to preclude further degradation of Ang I. The results showed that AGT, but not renin, was present in pseudocyst fluid at concentrations lower than circulating levels but higher than in CSF. However, the ratio of AGT to total proteins was much higher in the plasma than in pseudocyst fluid, suggesting that the AGT in pseudocyst does not result from simple diffusion from plasma. Thus, the information obtained using these approaches indicated that the upstream components of the RAS are expressed in tumour cells of human glioblastoma.

**Figure 3:**
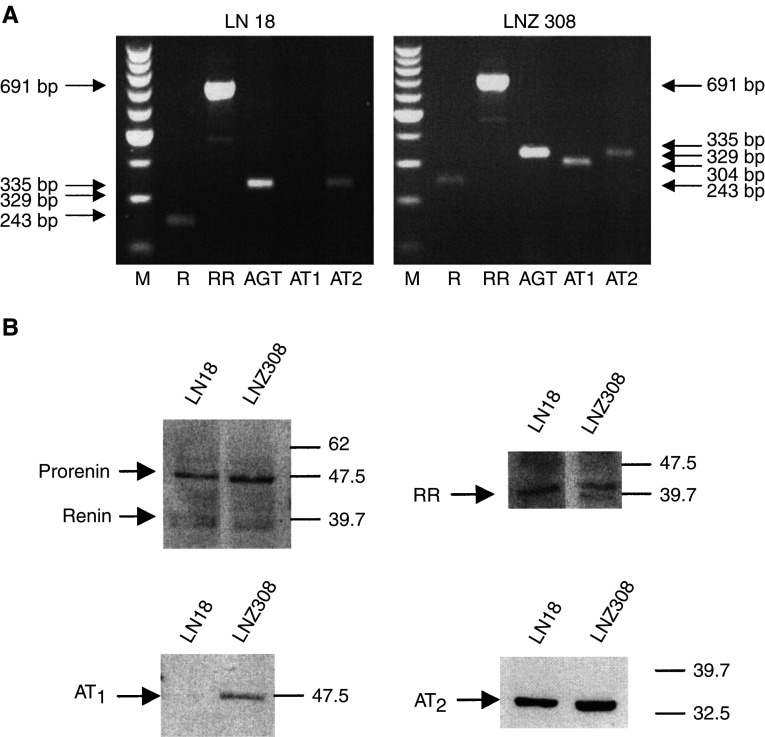
Determination of renin, renin receptor, AGT, AT_1_ and AT_2_ in human LN18 and LNZ308 glioblastoma cells. (**A**) RNA extracted from human glioblastoma cell lines were used for RT–PCR amplification of renin (R), renin receptor (RR), AGT and angiotensin receptor (AT_1_ and AT_2_) mRNAs, using the specific primers described in Table 1. (**B**) Proteins extracted from human glioblastoma cell lines were used for Western blotting analysis of the expression of renin, RR, AT_1_ and AT_2_ proteins.

Having established the presence of most of the RAS components in human glioblastoma, we addressed its role in tumour cells using the human LN18 and LNZ308 glioblastoma cell lines (Diserens *et al*, 1981; Egidy *et al*, 2000; Juillerat-Jeanneret *et al*, 2000). Both cell lines expressed renin, renin receptor, AGT and AT_2_ mRNAs using RT–PCR, while only LNZ308 expressed AT_1_ mRNA (Figure 3A

). Using Western blotting, renin (mainly produced as prorenin), renin receptor and AT_2_ proteins were expressed in both cell lines, while only LNZ308 cells expressed AT_1_ protein, corresponding to mRNA expression (Figure 3B). We had previously shown using an IRMA immunoassay (Juillerat-Jeanneret *et al*, 2000) that renin protein can be measured in cell extracts of human glioblastoma cells, but not in the culture supernatants. We have also previously shown that ACE activity is not measurable in glioblastoma cells in culture (Juillerat-Jeanneret *et al*, 2000). Together, these results suggest that human glioblastoma cells have the potential to produce AGT, prorenin/renin and Ang I, but not to convert Ang I into Ang II.

**Figure 4:**
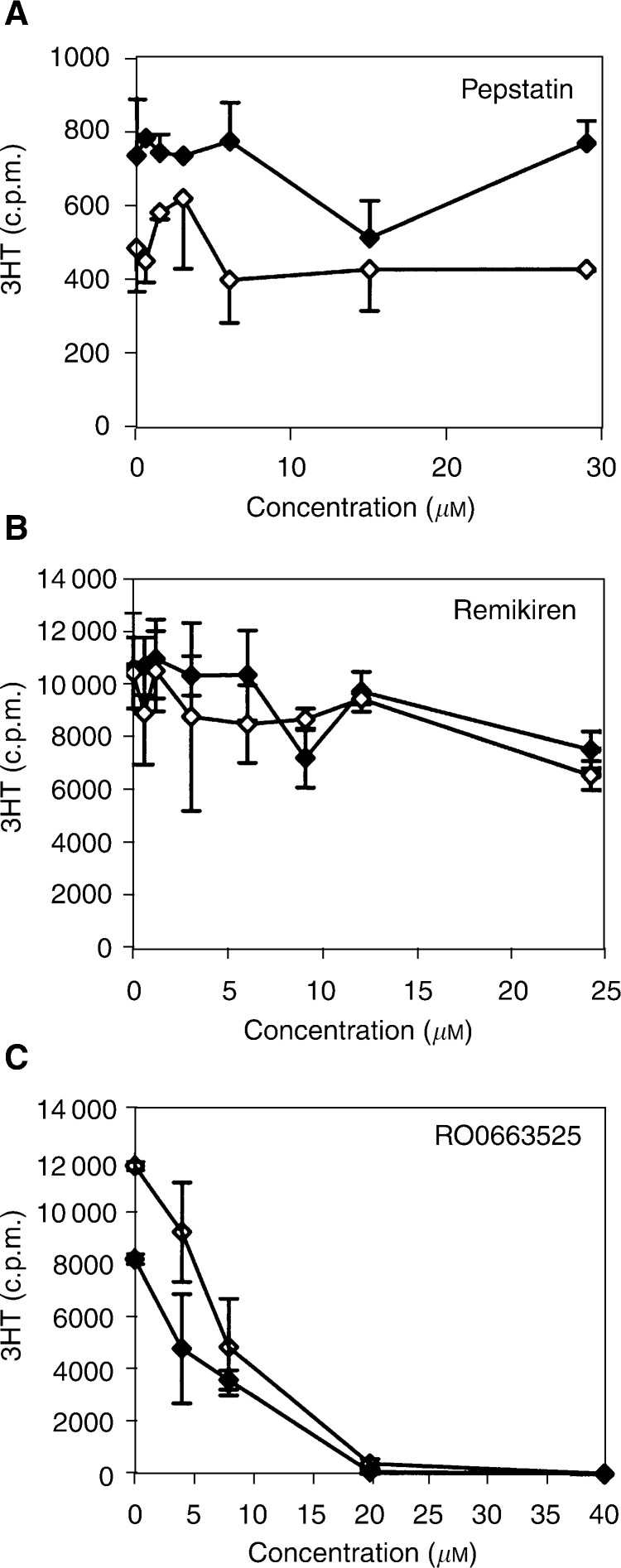
Renin inhibitors selectively decrease DNA synthesis in human glioblastoma cells. Cells were grown to confluence in culture medium containing FCS, then exposed for 24 h to different renin inhibitors at increasing concentrations in fresh medium containing FCS. ^3^H-thymidine (^3^HT) incorporation to quantify DNA synthesis was performed during the two last hours of incubation. (**A**) Pepstatin; (**B**) remikiren; (**C**) RO0663525. ♦: LN18 cells; ⋄: LNZ308 cells. Means±s.d. were calculated.

To address the functions of the RAS in human glioblastoma, we first evaluated the effect of inhibiting renin and ACE in glioblastoma cells. Three renin inhibitors, pepstatin, remikiren and RO0663525, at increasing concentrations were used to evaluate for the functions of renin in glioblastoma cells. While pepstatin and remikiren had little effect on glioblastoma cells, the addition of the RO0663525 synthetic renin inhibitor to tumour cells abolished DNA synthesis after 9 h or 24 h (Figure 4

) (Figure 5A

**Figure 5:**
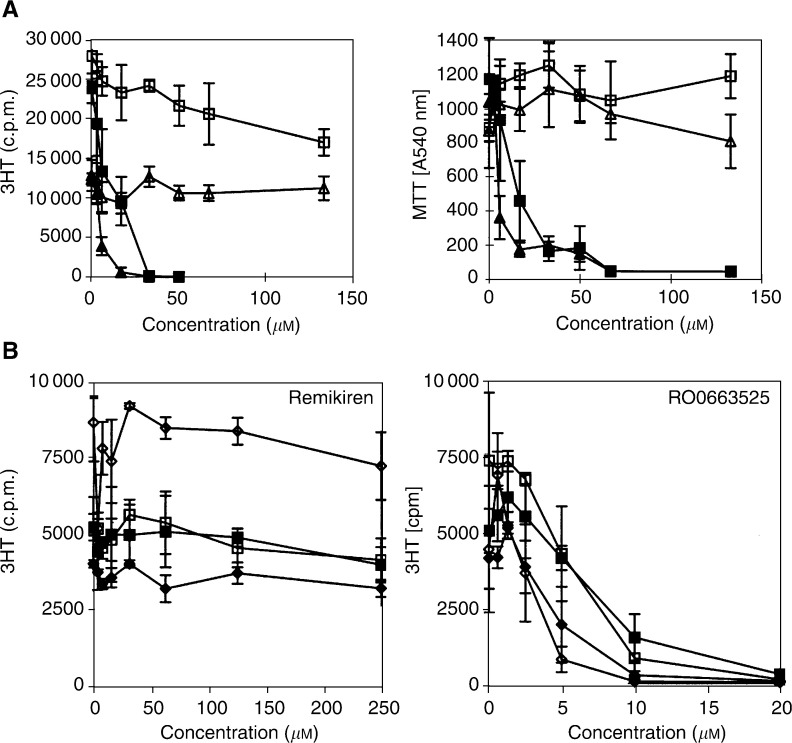
Exposure to RO0663525, but not to remikiren, results in inhibition of DNA synthesis and, concomitantly, a decrease of metabolically active viable glioblastoma cells. (**A**) Cells were grown to confluence in culture medium containing FCS, then exposed to renin inhibitors at increasing concentrations in fresh medium containing FCS either for 9 h and ^3^H-thymidine (^3^HT) incorporation to quantify DNA synthesis was performed for the last two hours of incubation, or for 24 h and the number of metabolically active cells was determined using a MTT assay for the last two hours. LN18: ▴: RO0663525; ▵: remikiren; LNZ308: ▪: RO0663525; □ : remikiren. (**B**) Cells were grown to confluence in culture medium containing FCS, then either deprived of FCS for 24 h or maintained in FCS-containing medium, and exposed to Remikiren or RO0663525 renin inhibitors at increasing concentrations in fresh medium containing or not FCS for 9 h and ^3^H-thymidine (^3^HT) incorporation to quantify DNA synthesis was performed for the last two hours of incubation. ♦: LN18 cells with FCS; ⋄: LNZ308 cells no FCS; ▪: LNZ308 cells with FCS; □: LNZ308 cells no FCS.

), as well as decreased the number of viable tumour cells after 24 h (Figure 5A). The presence of FCS did not modify glioblastoma cell response to the renin inhibitors (Figure 5B). The effect of the RO0663525 renin inhibitor on DNA synthesis was rapid, since identical inhibition was obtained after 2 or 8 h exposure, indicating that new protein synthesis was not necessary to obtain this effect (Figure 6

**Figure 6:**
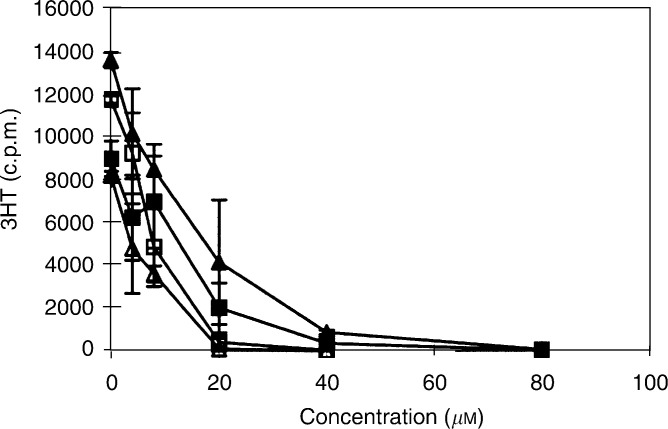
Inhibition of DNA synthesis by RO0663525 is an immediate effect. Cells were grown to confluence in culture medium containing FCS, then exposed for 2 h or 8 h to RO0663525 renin inhibitor at increasing concentrations in fresh medium containing FCS. ^3^H-thymidine (^3^HT) incorporation to quantify DNA synthesis was performed during the last two hours of incubation. LN18: ▴: 2 h; ▵: 8h; LNZ308: ▪: 2 h, □ 8 h. Means±s.d. were calculated.

). RO0663525 renin inhibitor induced apoptosis in glioblastoma cells after 9 h exposure, but only in cells previously deprived of FCS for 24 h (Figure 7A and B

**Figure 7:**
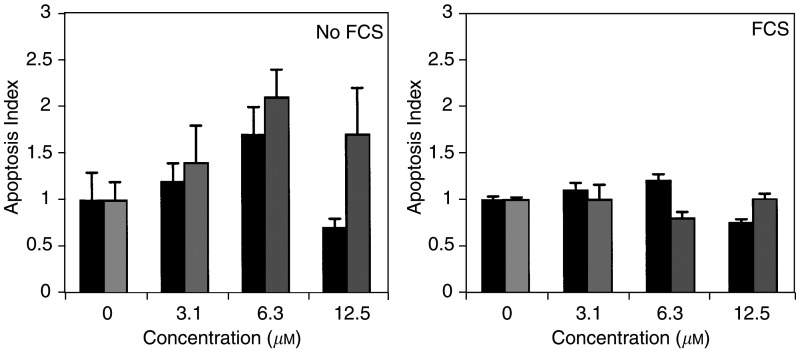
Induction of apoptosis by RO0663525. Cells were grown to confluence in culture medium containing FCS, then either deprived of FCS for 24 h (**A**) or maintained in medium with FCS (**B**), exposed for 9 h to RO0663525 renin inhibitor at increasing concentrations in fresh medium without FCS, and apoptosis index was determined by quantification of nucleosome fragments. Black bars: LN 18 cells; grey bars: LNZ308 cells. Means±s.d. were calculated.

). The ACE inhibitors captopril and lisinopril did not modulate cell proliferation after 24 or 48 h (results not shown).

**Figure 8:**
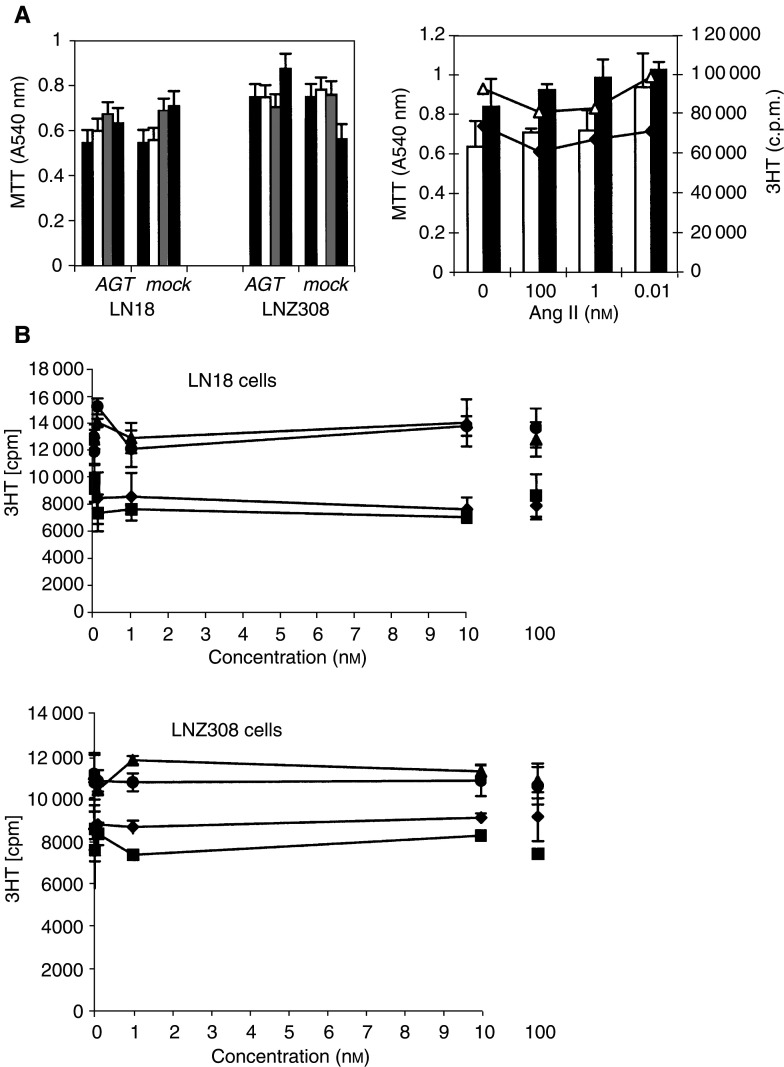
Angiotensinogen, tetradecapeptide renin substrate (Ang1-14), Ang I, Ang II or Ang III do not modulate glioblastoma cell growth. (**A**) *Left panel*: Confluent cultures of LN18 and LNZ308 cells were deprived of FCS for 24 h, then exposed for 48 h in the absence of FCS either to 1–10% of AGT-containing-CHO or mock-transfected cell supernatants in DMEM (black bars: 0%, white bars: 1%, 0.15 *μ*g AGT ml^−1^, light grey bars: 5%, 0.75 *μ*g AGT ml^−1^, dark grey bars: 10%, 1.5 *μ*g AGT ml^−1^). *Right panel:* Confluent cultures of LN18 and LNZ308 cells were deprived of FCS for 24 h, then either exposed for 7 h to 10^−7^, 10^−9^ or 10^−11^ M Ang II, and thymidine incorporation was performed for 2 h to determine DNA synthesis (^3^HT; ♦: LN18; ▵: LNZ308) or exposed for 24 h to 10^−11^, 10^−9^ or 10^−7^ M Ang II, and MTT reduction was performed to determine the number of metabolically active viable cells (MTT; white bars: LN18; grey bars: LNZ308). Means±s.d. were calculated. (**B**) LN18 and LNZ308 cells were grown for 24 h to half-confluence in the presence of FCS, then deprived of FCS for 24 h, and exposed for another 24 h in the absence of FCS to increasing concentration [0–100 nM] of either tetradecapeptide renin substrate (Ang1–14) (♦), Ang I (▪), Ang II (▴), or Ang III (•) for 24 h. Thymidine (^3^HT) incorporation was performed during the last two hours to determine DNA synthesis (^3^HT). Means±s.d. were calculated.

Then, we evaluated whether the production of angiotensin peptides was involved in glioblastoma cell growth, survival and/or apoptosis. Neither AGT nor Ang II modulated glioblastoma cell growth, as determined by thymidine incorporation to quantitate DNA synthesis and MTT reduction to quantitate metabolically active cells (Figure 8A

). Tetradecapeptide renin substrate (TDP/AGT1-14), Ang I or Ang III comparably to Ang II, even at high concentration, did not modulate thymidine incorporation (Figure 8B). AGT did not induce apoptosis or sensitise LN18 and LNZ308 cells to FasL-induced apoptosis (Table 3

**Table 3:** Angiotensinogen neither induces apoptosis, nor inhibits or sensitises glioblastoma cells to FasL-induced apoptosis

	**Apoptosis index**			
	**LN18**		**LNZ308**	
**Treatment% of CHO SN (*μ*g AGT ml^−1^)**>	**No FasL**	**2% FasL**	**No FasL**	**2% FasL**
*AGT-CHO cells*				
0% (0 *μ*g AGT ml^−1^)	1.00	3.55	1.00	1.48
1% (0.15 *μ*g ml^−1^)	1.52	2.65	1.35	2.19
5% (0.75 *μ*g ml^−1^)	2.01	3.92	2.39	1.74
10% (1.50 *μ*g ml^−1^)	1.79	4.88	1.80	1.93

*Mock-transfected-CHO cells*				
0%	1.00	3.55	1.00	1.48
1%	1.66	4.74	1.54	2.03
5%	1.91	5.38	0.96	1.92
10%	1.31	3.28	1.72	2.38

AGT: angiotensinogen. LN18 or LNZ308 cells were treated for 24 h in the absence of FCS either with 1–10% of angiotensinogen-containing supernatant of AGT-transfected CHO cells (0–1.5 *μ*g AGT ml^−1^) or mock-transfected cells, or 2% of FasL-containing supernatant of Neuro2A-FasL cells or a combination of both. At the end of the incubation period, apoptosis index was quantified by measurement of nucleosome fragments in cells. Means of duplicate wells were calculated.

). We had previously shown (Egidy *et al*, 2000) that LN18 cells are sensitive to FasL-induced apoptosis, while LNZ308 cells needs sensitisation to undergo FasL-induced apoptosis. Thus, neither AGT, renin, TDP, Ang I, nor Ang II, the active hormone of the systemic RAS, nor Ang III, the predominant peptide produced in human glioblastoma (Juillerat-Jeanneret *et al*, 2000), are important in directly regulating glioblastoma cell growth and/or controlling apoptosis. Addition of the AT_2_ antagonist PD123319 to glioblastoma cells expressing this receptor did not modify DNA synthesis (results not shown). Exogenous addition of Ang II or AT_1_ or AT_2_ receptor antagonists together with renin inhibitors either did not counterbalance the growth-inhibitory effects RO0663525 or did not potentiate Remikiren effects (results not shown). Thus, the active molecule of the RAS in glioblastoma cells is the enzyme prorenin/renin, independently of the binding of angiotensin peptides to their cell-membrane receptors.

## DISCUSSION

The expression of renin, AGT, ACE and AT_1_ receptor in the developing and normal brain has been previously described. In normal brain, the main cells expressing AGT are mostly astrocytes (Humpel *et al*, 1994), while renin is expressed both by astrocytes and neuron populations. However, most information has been obtained in rodents. Renin expression has been previously demonstrated by immunohistochemistry in human glioblastoma (Ariza *et al*, 1988) and AGT synthesis by Northern blotting in nontumoral (*n*=3) and tumoral (*n*=3) astrocytic cell lines (Milsted *et al*, 1990). In this small series, AGT expression was higher in normal than tumoral cells, with heterogenous expression between cells. No expression of renin or ACE was found. ACE is ubiquitously expressed by the vascular system including brain vasculature. We have previously shown that human glioblastoma cell lines did not express ACE activity, but that ACE is highly expressed in the abnormal vessels of human glioblastoma (Juillerat-Jeanneret *et al*, 2000). From this information, it was postulated that a complete angiotensin system existed in the brain, independently of the circulating system, and it was assumed that its role, including in tumours, was important in the regulation of vascular functions. In the vasculature, AGT, the unique and specific substrate of renin, was demonstrated to be antiangiogenic (Célérier *et al*, 2000, 2002), while angiotensin peptides, in particular Ang II, were shown to be proangiogenic and to be involved in vascular growth (Rivera *et al*, 2001). However, no study has directly questioned the functions of the RAS, and of its components, in tumoral astrocytes such as human glioblastoma cells.

In cancer, the role of the RAS has been mainly evaluated in the context of vascular functions (Achard *et al*, 2001). In a C6 glioblastoma rat model, losartan (an AT_1_ antagonist) reduced tumour growth, vascular density, tumour cell proliferation and mitotic index (Rivera *et al*, 2001). In the present study, we demonstrate that AGT, prorenin, ACE, AT_1_ and AT_2_ are synthesised and expressed in human glioblastoma and glioblastoma cells in culture, however at different levels of expression between the specimens. Glioblastoma are highly heterogeneous tumours. Our results suggest that the expression of the RAS components varies according to the particular glioblastoma and/or tumour area, and thus reflects the clonal heterogeneity of glioblastoma. AGT is released in the tumour pseudocyst *in vivo* in humans, and ACE is expressed by tumour-associated vasculature, suggesting a potential production of all RAS components in the tumour environment.

We addressed first the functions of the angiotensin peptides in glioblastoma cells. In order to exclude an indirect effect of Ang peptides transactivating other signaling pathways, we only studied the effects of these peptides for short time exposure. We showed that AGT, TDP renin substrate and Ang peptides did not play any role in glioblastoma cell growth, apoptosis and/or DNA synthesis. It may be hypothesised from previous information that their targets, if these peptides are produced by glioblastoma cells, is the tumour-associated vasculature. These effects involve AT_1_ receptors, while AT_2_ receptors may be growth inhibitory and pro-apoptotic (Berry *et al*, 2001). AGT and des(Ang I)-AGT have been shown to be anti-angiogenic (Célérier *et al*, 2002). Immunohistochemistry for AGT performed on normal tissue, and grade II and III astrocytoma and glioblastoma multiform (grade IV) suggested that the expression of this protein was inversely related to tumour grading (manuscript in preparation).

We then addressed the effects of inhibiting the enzymes of the RAS in human glioblastoma. ACE inhibitors may be of some benefit in cancer (Nakagawa *et al*, 1995; Reddy *et al*, 1995; Lever *et al*, 1998; Yoshiji *et al*, 2002), mainly acting via their antiangiogenic potential and as more general zinc metalloprotease inhibitors, independently of the RAS and of ACE inhibition. Our previous experiments with lisinopril and experimental glioblastoma in the rat did not show any benefit in glioblastoma (Juillerat-Jeanneret *et al*, 2000); however, we cannot exclude that lisinopril was not transported across the cerebral vasculature (the blood–brain barrier), which is not as leaky in experimental rodent models as in human glioblastoma. In the present study of human glioblastoma cells in culture, we did not show any effect of the ACE-inhibitors captopril and lisinopril in cancer cell growth and DNA synthesis, showing the specificity of ACE inhibitors between tumour cells of different origins.

However, one renin-selective inhibitor could induce a rapid and important blockade of DNA synthesis, apoptosis and loss of viable cells in human glioblastoma cells in culture. This effect of renin inhibitors seems to depend on the lipophilicity, efficacy (IC_50_/*K*_i_) and chemical structure of the compounds, since RO0663525, a nonpeptidomimetic representative of the piperidine class of inhibitors (Güller *et al*, 1999; Oefner *et al*, 1999; Maerki *et al*, 2001), but not the more hydrophilic peptidomimetics remikiren and pepstatin, was efficient. Pepstatin is a better inhibitor of cathepsin D than of renin (Baldwin *et al*, 1993). Cathepsin D is more frequently expressed in carcinomas than in connective tissue neoplasm such as glioblastoma (Reid *et al*, 1986); thus an inhibitory effect on cathepsin D can be excluded. We have previously shown that human glioblastoma cells do not secrete renin (Juillerat-Jeanneret *et al*, 2000). Thus, the inhibitory pattern of renin inhibitors suggests an intracellular function of prorenin/renin, and thus only hydrophobic inhibitors have the potential to inhibit intracellular renin. It has been shown that an alternative transcription site for renin may be used in the brain, which would result, if transcribed, in the production of an altered form of prorenin mRNA, not secreted and constitutively active (Lee-Kirch *et al*, 1999; Sinn and Sigmund, 2000). Alternatively, it has been shown that the binding of piperidine inhibitors to the renin active site pocket results in an induced structural fit of the enzyme, which has not been demonstrated for peptidomimetic inhibitors (Deinum *et al*, 1998; Vieira *et al*, 1999; Maerki *et al*, 2001). This conformational change may modify the binding of renin/prorenin to its recently described renin receptor (Nguyen *et al*, 2002). We show here that human glioblastoma and glioblastoma cells in culture express and synthesise the renin receptor. This hormone-like function of renin/prorenin activates the intracellular signaling ERK pathways, and survival signalling. We have previously shown that endothelin receptor antagonists sensitise glioblastoma cells to Fas-L-induced apoptosis involving the ERK pathway and the regulation of anti/proapoptotic molecules (Egidy *et al*, 2000), raising the possibility of a comparable role for renin/renin-receptor functions, since we show here that RO0663525 can induce apoptosis in human glioblastoma cells.

In conclusion, our results suggest that the expression of the RAS in glioblastoma is heterogeneous and that its functions are double. First, AGT secreted by astrocytes, whether normal or tumoral cells, and neuron-derived and vascular-derived renin, ACE and aminopeptidases will produce angiotensin peptides (Ang I, Ang II, Ang III). It can be hypothesised from previous information that these peptides acting through angiotensin receptors on tumour-associated vasculature, regulate vascular functions and angiogenesis. Second, we have shown that renin has a direct role in glioblastoma cell proliferation and/or survival and that inhibitors of the enzyme induce a fast and important loss of proliferation and survival of glioblastoma cells, independent of the action of angiotensin peptides on their cognate receptors. Two models may explain this effect: (1) by inducing conformational changes, renin inhibitors abolish the function of renin on its receptor, activation of the receptor and intracellular survival signaling; (2) the functions of renin in glioblastoma cells may be intracellular, and do not involve cell-membrane-bound angiotensin receptors. These issues need to be resolved, but renin inhibition, in combination therapy with other drugs, may be a potential approach to control glioblastoma progression.
